# Applicability of predictive toxicology methods for monoclonal antibody therapeutics: status Quo and scope

**DOI:** 10.1007/s00204-016-1876-7

**Published:** 2016-10-20

**Authors:** Arathi Kizhedath, Simon Wilkinson, Jarka Glassey

**Affiliations:** 1 Chemical Engineering and Advanced Materials, Newcastle University, Newcastle upon Tyne, NE17RU UK; 2 Medical Toxicology Centre, Institute of Cellular Medicine, Newcastle University, Newcastle upon Tyne, NE2 4AA UK

**Keywords:** Predictive toxicology, Monoclonal antibody-based therapeutics, Safety pharmacology, In vitro and in silico tools, QSAR

## Abstract

Biopharmaceuticals, monoclonal antibody (mAb)-based therapeutics in particular, have positively impacted millions of lives. MAbs and related therapeutics are highly desirable from a biopharmaceutical perspective as they are highly target specific and well tolerated within the human system. Nevertheless, several mAbs have been discontinued or withdrawn based either on their inability to demonstrate efficacy and/or due to adverse effects. Approved monoclonal antibodies and derived therapeutics have been associated with adverse effects such as immunogenicity, cytokine release syndrome, progressive multifocal leukoencephalopathy, intravascular haemolysis, cardiac arrhythmias, abnormal liver function, gastrointestinal perforation, bronchospasm, intraocular inflammation, urticaria, nephritis, neuropathy, birth defects, fever and cough to name a few. The advances made in this field are also impeded by a lack of progress in bioprocess development strategies as well as increasing costs owing to attrition, wherein the lack of efficacy and safety accounts for nearly 60 % of all factors contributing to attrition. This reiterates the need for smarter preclinical development using quality by design-based approaches encompassing carefully designed predictive models during early stages of drug development. Different in vitro and in silico methods are extensively used for predicting biological activity as well as toxicity during small molecule drug development; however, their full potential has not been utilized for biological drug development. The scope of in vitro and in silico tools in early developmental stages of monoclonal antibody-based therapeutics production and how it contributes to lower attrition rates leading to faster development of potential drug candidates has been evaluated. The applicability of computational toxicology approaches in this context as well as the pitfalls and promises of extending such techniques to biopharmaceutical development has been highlighted.

**Electronic supplementary material:**

The online version of this article (doi:10.1007/s00204-016-1876-7) contains supplementary material, which is available to authorized users.

## Introduction

The pharmaceutical industry is currently valued at $786 billion from the total worldwide sales of prescription as well as over the counter drugs in 2015 wherein 25 % of this revenue was generated by biological/biotechnological products (Pharma 2014). Biological drugs are associated with living entities (cells and tissues) and/or their product such as recombinant therapeutic proteins and vaccines to name a few. Based on historical data, a shift towards biologics seems imminent owing to increasing profits and lower attrition rates when compared to small molecule drugs. Biological drugs comprised 70 % of the top ten selling products of the world in 2014, and the percentage sales of biotechnology products within the top 100 was 44 %. Twenty new biologicals were approved by FDA in 2014 compared to the 11 that were approved in 2009. Monoclonal antibodies have higher approval rates of 26 % in the biopharmaceutical sector than that of conventional small molecule drugs (10 %) (Hay et al. 2014). Based on the area of therapy, the largest segments of oncology and anti-rheumatoid drugs, which contribute to a combined compound annual growth rate of 13 %, continue to be dominated by biological drugs.

**Fig. 1:**
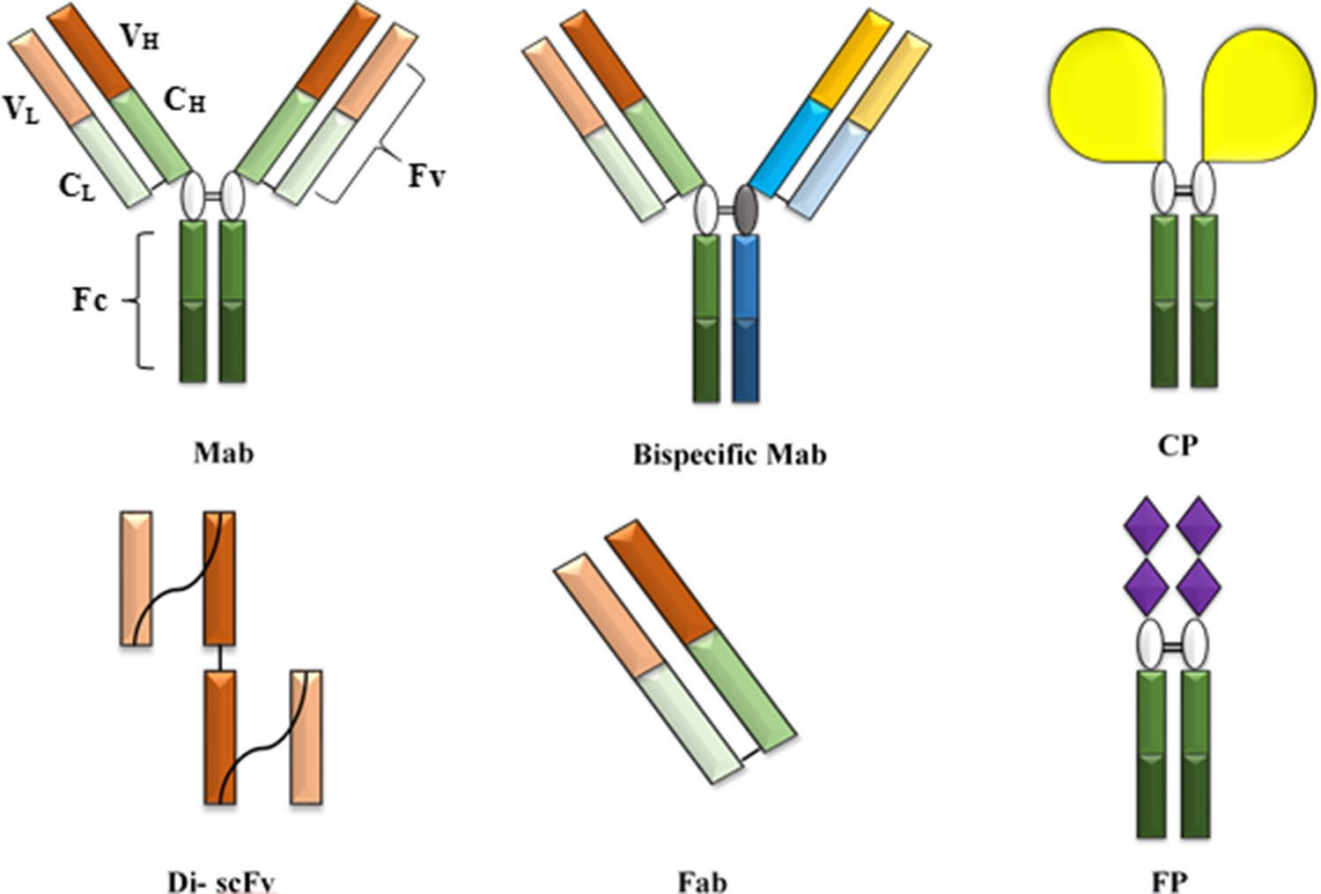
Generic monoclonal antibody-derived therapeutic structures as adapted from IMGT (Lefranc et al. 2009; World Health O 2006). *Fc* constant region which contributes to effector function, immune response and increased half-life, *Fv* variable region that contains complementarity determining regions (CDRs) facilitating antigen binding, *Fab* antigen binding fragment which lacks Fc region, *scFv* single chain fragment variable, *FP* Fc fusion proteins that contain Fc region for effector functionality (e.g. Abatacept), *CP* composite protein that contains Fc region for increasing half-life and not for effector functionality (e.g. Strensiq™) (World Health 2006)

Even though the therapeutic efficiency of immunoglobulin molecules was demonstrated in 1890, it was only after Kohler and Milstein elucidated the murine hybridoma technology for in vitro production of mAbs (see Fig. 1 for generic mAb structures) that the market for mAbs grew and expanded to different therapy areas, such as haematology, oncology, immunology, cardiology, infectiology and ophthalmology as well as diagnostics and imaging(Köhler and Milstein 1975). The shift from murine mAbs to chimeric (human Fc region with murine Fv region) was mainly to increase titres as well as decrease immunogenic effects (Zhu 2012). To further decrease the murine composition and enhance Fc functionality, humanized mAbs were first developed in 1986 (Jones et al. 1985). The production systems routinely used for chimeric and humanized mAbs are Chinese hamster ovary (CHO) cells, NS0 and Sp2/0 myeloma cell line. To fully eliminate the immunogenic potential of murine epitopes while maintaining optimal Fc region functionality, fully human mAbs were developed by phage display technology and commercially produced by CHO system (Lai et al. 2013). Human embryonic kidney (HEK) and human retinal cell-derived (Per.C6) cell lines are the new potential candidates for biopharmaceutical production (Zhu 2012). In addition to being stable and producing high titres, the fully human cell lines offer the advantage of proper post-translation modification and glycosylation as they incorporate human biosynthetic pathways. Plant expression systems, such as recombinant *Agrobacterium tumefaciens,* and microbial systems, such as *Escherichia coli,* are gaining popularity for production of monoclonal antibodies against viruses (Berlec and Štrukelj 2013; Rosenberg et al. 2013; Ma et al. 2003). Transfected HEK cells have already been used to produce recombinant coagulation factors which have been approved by FDA (Food and Drug Administration); however, full length mAbs produced by them are still awaiting approval (Lai et al. 2013; Berlec and Štrukelj 2013). Furthermore proprietary technologies, such as VelocImmune^®^, BiTE^®^, POTELLIGENT™, UltiMAb^®^ and XenoMouse^®^, are used for production of monoclonal antibodies (Jakobovits et al. 2007; Murphy 2009; Nelson and Paulos 2015; Sheridan 2010; Shitara 2009). The mAb-derived products include fusion proteins, antigen binding fragments as well as composite proteins (Lefranc et al. 2009; Povey et al. 2001; Ecker et al. 2015; Li and Zhu 2010).

### MAbs: safety pharmacology and side effects

MAbs and related therapeutics are highly desirable from a biopharmaceutical perspective as they are highly target specific and well tolerated within the human system. Nevertheless, several mAbs have been discontinued or withdrawn based either on their inability to demonstrate efficacy and/or due to adverse effect, for example, Efalizumab, Biciromab and Fanolesomab, while others were discontinued due to high manufacturing costs, for example, Imciromab and Arcitumomab (Lefranc et al. 2009). Approved monoclonal antibodies as well as derived products have been associated with adverse effect, and these effects have been classified into categories of specialized toxicity as indicated in Table 1 (Peluso et al. 2013; Hansel et al. 2010). The reporting of these adverse effects is to be treated with caution as there are several factors that influence them, such as underlying conditions, drug combinations, reporting practices and clinical practice involved in the clinical trials.

**Table 1:** List of approved monoclonal antibody-derived therapeutics and toxicity

Generic name Trade name	Type	Antigen^a^	Species	Therapy area	Production	Therapy-associated toxicity
Abatacept ORENCIA^®^	FP	CD80, CD86	*Homo sapiens*	Immunology	CHO	Occular toxicity, immunotoxicity, dermal toxicity, infection
Abciximab REOPRO^®^	Fab IgG1κ	ITGA2B_ITGB3	Chimeric	Cardiology	Sp2/0	Immunotoxicity, haemotoxicity
Adalimumab HUMIRA^®^	IgG1κ	TNF	*Homo sapiens*	Immunology	CHO	Immunotoxicity cardiotoxicity, infection, hepatoxicity haemotoxicity, others
Aflibercept ZALTRAP^®^ EYLEA^®^	FP	VEGFA	*Homo sapiens*	Ophthalmology, oncology	CHO K-1	Occular toxicity haemotoxicity cardiotoxicity
Alemtuzamab CAMPATH-1H^®^, LEMTRADA^®^	IgG1κ	CD52	Humanized	Haematology, oncology, immunology	CHO	Immunotoxicity, haemotoxicity cardiotoxicity others
Alirocumab PRALUENT^®^	IgG1κ	PCSK9	*Homo sapiens*	Cardiology	VelocImmune^®^	Neurotoxicity, dermal toxicity occular toxicity cardiotoxicity
Asfotase alpha STRENSIQ™	CP		*Homo sapiens*	Hypophosphatas-ia	CHO	Immunotoxicity dermal toxicity renal toxicity, occular toxicity others
Basiliximab SIMULECT^®^	IgG1κ	IL2RA	Chimeric	Immunology	Sp2/0	Immunotoxicity dermal toxicity
Belatacept NULOJIX^®^	FP	CD80, CD86	*Homo sapiens*	Immunology	CHO	Renal toxicity, infection, others
Belimumab BENLYSTA^®^	IgG1λ	TNFSF13B	*Homo sapiens*	Immunology	NS0 (serum free)	Immunotoxicity infection, others
Besilesomab SCINTIMUN^®^	IgG1κ	CEACAM8	*Mus musculus*	Osteology (diagnostic)	Hybridoma technology*	Cardiotoxicity immunotoxicity
Bevacizumab AVASTIN^®^	IgG1κ	VEGFA	Humanized	Oncology	CHO	Cardiotoxicity, infection, haemotoxicity, gastrointestinal, others
Blinatumomab BLINCYTO^®^	scFv κH–scFv κH	CD19, CD3E	*Mus musculus*	Haematology, oncology	BiTE^®^	Immunotoxicity, neurotoxicity
Brentuximab ADCETRIS™	IgG1κ	TNFRSF8	Chimeric	Oncology	CHO	Cardiotoxicity, infection, pulmonary toxicity
Canakinumab ILARIS^®^	IgG1κ	IL1B	*Homo sapiens*	Hereditary inflammatory diseases; immunology	UltiMAb^®^	Infection, others
Capromab PROSTASCINT^®^	IgG1κ	FOLH1	*Mus musculus*	Oncology	Hybridoma technology**	NR
Catumaxomab REMOVABT^®^	IgG2aκ/G2bλ	CD3E, EPCAM	*Mus musculus* *Rattus sp*. Hybrid	Oncology	Quadroma technology^+^	Haemotoxicity, immunotoxicity, others
Certolizumab CIMZIA^®^	Fab´-G1κ	TNF	Humanized	Immunology	*Escherichia coli*	Immunotoxicity cardiotoxicity, infection, hepatoxicity haemotoxicity
Cetuximab ERBITUX^®^	IgG1κ	EGFR	Chimeric	Oncology	Sp2/0	Immunotoxicity, dermal toxicity, pulmonary toxicity
Daclizumab*** ZENAPAX^®^	IgG1κ	IL2RA	Humanized	Immunology	NS0	Immunotoxicity, dermal toxicity
Daratumumab DARZALEX™	IgG1κ	CD38	*Homo sapiens*	Haematology, oncology, immunology	UltiMAb^®^	Haemotoxicity, immunotoxicity, pulmonary toxicity
Denosumab PROLIAS^®^ XGEVAS^®^	IgG2	TNSF11	*Homo sapiens*	Osteology	XenoMouse^®^	Haemotoxicity, infection
Eculizumab SOLIRIS™	IgG2/G4κ	C5	Humanized	Haematology	NS0	Haemotoxicity, infection
Edrecolomab PANOREX^®^	IgG2aκ	EPCAM	*Mus musculus*	Oncology	Sp2/0	Immunotoxicity, others
Elotuzumab EMPLICITI™	IgG1κ	SLAMF7	Humanized	Haematology, oncology, immunology	NS0 (Varma et al. 2014)	Haemotoxicity, gastrointestinal, others
Etanercept ENBREL^®^	FP	TNF	*Homo sapiens*	Immunology	CHO	Infection, cardiotoxicity, hepatotoxicity, immunotoxicity
Evolocumab REPATHA™	IgG2λ	PCSK9	*Homo sapiens*	Cardiovascular diseases	XenoMouse^®^	Immunotoxicity, haemotoxicity, infection, others
Factor IX Fc FP ALPROLIX^®^	CP	NA	*Homo sapiens*	Haematology	Transfected HEK cell line	NR
Factor VIII Fc FP ELOCTATE^®^	CP	NA	*Homo sapiens*	Haematology	Transfected HEK cell line.	NR
Golimumab SIMPONI^®^ SIMPONI ARIA^®^	IgG1κ	TNF	*Homo sapiens*	Immunology	UltiMAb^®^	Dermal toxicity
Ibritumomab ZEVALIN^®^	IgG1κ	MS4A1	*Mus musculus*	Oncology	CHO	Haemotoxicity, dermal toxicity, others
Idarucizumab PRAXBIND^®^	Fab-G1κ	Pradaxa^®^: Dabigatran etexilate mesylate	Humanized	Reversal of drug overdose	CHO	Dermal toxicity, gastrointestinal, infection, others
Infliximab REMICADE^®^	IgG1κ	TNF	Chimeric	Immunology	Sp2/0	Immunotoxicity cardiotoxicity, infection, hepatoxicity haemotoxicity, others
Ipilimumab YERVOY^®^	IgG1κ	CTLA4	*Homo sapiens*	Oncology	UltiMAb^®^	Hepatotoxicity, neurotoxicity, pulmonary toxicity, gastrointestinal toxicity
Mepolizumab NUCALA^®^	IgG1κ	IL5	Humanized	Immunology	CHO	Infection, cardiotoxicity, others
Mogamulizumab POTELIGEO^®^	IgG1κ	CCR4	Humanized	Haematology, oncology	POTELLIGENT^®^	Immunotoxicity, dermal toxicity
Muromonab-CD3 ORTHOCLONE OKT3^®^	IgG2aκ	CD3E	*Mus musculus*	Immunology	Hybridoma murine ascites	Immunotoxicity, hepatotoxicity, cardiotoxicity
Natalizumab TYSABRI^®^	IgG4	ITGA4	Humanized	Immunology	NS0	Immunotoxicity, hepatotoxicity, infection
Necitumumab PORTRAZZA™	IgG1κ	EGFR	*Homo sapiens*	Oncology	UltiMAb^®^	Haemotoxicity, immunotoxicity, pulmonary toxicity, hepatotoxicity
Nimotuzumab THERACIM^®^	IgG1κ	EGFR	Humanized	Oncology	NS0	Dermal toxicity
Nivolumab OPDIVO^®^	IgG4κ	PDCD1	*Homo sapiens*	Oncology	UltiMAb^®^	Immunotoxicity, hepatotoxicity, gastrointestinal toxicity, pulmonary toxicity, renal toxicity
Obinutuzumab GAZYVA^®^	IgG1κ	MS4A1	Humanized	Haematology, oncology	GlycoMAb^®^	Infection
Ofatumumab ARZERRA^®^	IgG1κ	MS4A1	*Homo sapiens*	Haematology, oncology	UltiMAb^®^, NS0	Infection, gastrointestinal toxicity
Omalizumab XOLAIR^®^	IgG1κ	IGHE	Humanized	Immunology	CHO	Immunotoxicity, dermal toxicity, infection
Palivizumab SYNAGIS	IgG1κ	RSV glycoprotein F	Humanized	Infectiology	NS0	Immunotoxicity, others
Panitumumab VECTIBIX^®^	IgG2κ	EGFR	*Homo sapiens*	Oncology	XenoMouse^®^ CHO	Immunotoxicity, pulmonary toxicity, dermal toxicity
Pembrolizumab KEYTRUDA^®^	IgG4κ	PDCD1	Humanized	Oncology	CHO	Immunotoxicity, pulmonary, others
Pertuzumab PERJETA^®^	IgG1κ	ERBB2	Humanized	Oncology	CHO^++^	Reproductive and developmental toxicity, dermal toxicity, haemotoxicity, immunotoxicity, cardiotoxicity
Ramucirumab CYRAMZA^®^	IgG1κ	KDR	*Homo sapiens*	Oncology	NS0	Haemotoxicity, cardiotoxicity, gastrointestinal, others
Ranibizumab LUCENTISO^®^	Fab G1κ	VEGFA	Humanized	Ophthalmology, immunology	*Escherichia coli*	Cardiotoxicity, haemotoxicity, occular toxicity
Raxibacumab ABTHRAX^®^	IgG1λ	Anthrax protective antigen	*Homo sapiens*	Infectiology	CHO	Haemotoxicity, infection, dermal toxicity, others
Rilonacept ARCALYSTF^®^	FP	IL1A	*Homo sapiens*	Immunology	CHO	Dermal toxicity, immunotoxicity
Rituximab MABTHERA^®^, RITUXAN^®^	IgG1κ	MS4A1	Chimeric	Haematology, oncology, immunology	CHO-MR	Immunotoxicity, cardiotoxicity, infection, others
Romiplostim NPLATE^®^	CP	MPL	*Homo sapiens*	Immunology	*Escherichia coli*	Haemotoxicity, infection, others
Secukinumab COSENTYX^®^	IgG1κ	IL17A	*Homo sapiens*	Immunology	XenoMouse^®^	Infection, haemotoxicity, cardiotoxicity
Siltuximab SYLVANT^®^	IgG1κ	IL6	Chimeric	Haematology, oncology, immunology	CHO	Immunotoxicity, gastrointestinal toxicity, infection
Tocilizumab ACTEMRA^®^ RoACTEMRA^®^	IgG1κ	IL6R	Humanized	Oncology, immunology	CHO-DR	Immunotoxicity, infection, hepatotoxicity, others
Trastuzumab HERCEPTIN^®^	IgG1κ	ERBB2	Humanized	Oncology	CHO-MR	Immunotoxicity, hepatotoxicity, cardiotoxicity, pulmonary toxicity, dermal toxicity
Ado-trastuzumab (emantsine) KADCYLAN^®^	IgG1κ	ERBB2	Humanized	Oncology	CHO	Reproductive and developmental toxicity, dermal toxicity, hepatotoxicity, cardiotoxicity
Ustekinumab STELARA^®^	IgG1κ	IL12B	*Homo sapiens*	Immunology	UltiMAb^®^	Neurotoxicity, cardiotoxicity others
Vedolizumab ENTYVIO^®^	IgG1κ	ITGA4 ITGB7	Humanized	Immunology	CHO	Infection, pulmonary toxicity, other

*FP* fusion protein, *CP* composite protein, *Fab* antigen binding fragment, *IgG* immunoglobulin G, *CHO* Chines hamster ovary cells, CHO-*DR* Chines hamster ovary cells dihydrofolate reductase; *CHO-MR* Chines hamster ovary cells methotrexate resistant; *NS0* non-secreting murine myeloma cells, *Sp2/0* hybridoma B lymphocyte, *NA* not applicable, *HEK* human embryonic kidney cell line

* X63Ag8.653 and spleen cells from Balb/c mice previously immunized with CEA antigen (from human liver metastasis)

** Fusing P3 × 63Ag8.653 myeloma cells with spleen cells from BALB/c mice immunized with whole cells and membrane extracts of the human prostate adenocarcinoma cell line LNCaP

*** EC withdrawal

^+^Consists of mouse IgG2a and rat IgG2b;^++^Fed-batch process using a suspension-adapted CHO cell line

^a^Nomenclature derived from HUGO Gene nomenclature Committee resources (Povey et al. 2001)

The catastrophic TGN1412 clinical trial that resulted in multiple organ failure of six healthy volunteers reiterated the need for better preclinical safety testing. The underlying problems that were subsequently identified in this trial were mainly the lack of appropriate preclinical testing and model organisms chosen for study of adverse effects. The standard in vitro assays failed to capture the in vivo adverse effects in humans (Stebbings et al. 2013). In vivo toxicity studies using rodent or primate models are not always representative of the human system. Human therapeutics such as monoclonal antibodies are highly specific and targeted, and there is, therefore, a higher likelihood of false positive efficacy or false negative toxicity if such entities are tested in non-human models, both outcomes being highly undesirable.

Eloctate showed haemotoxicity and hepatotoxicity in animal studies (mice and monkeys), but none have been reported in human clinical trials (Lower 2015). TGN1412 did not show the pro-inflammatory cytokine storm in in vivo tests (cynomolgus macaques) due to the absence of CD28 on its CD4^+^ effector memory T cells as well as in in vitro tests (human lymphocytes) due to the lack of localization of cell receptor (Stebbings et al. 2013). There are different factors which can influence the safety and efficacy of mAbs. Binding affinity, glycoforms, valency and density of antigens as well as antibodies, cell surface receptor and binding interface are some of the factors that contribute to the biological activity of mAbs and, if suboptimal, could lead to reduction of efficacy or an increase in toxicity (Stebbings et al. 2013; Jefferis 2014). Nimotuzumab exhibits lower dermal toxicity due to optimal binding affinity to EFGR that ensures its binding below toxic levels (Boland and Bebb 2009).

**Fig. 2:**
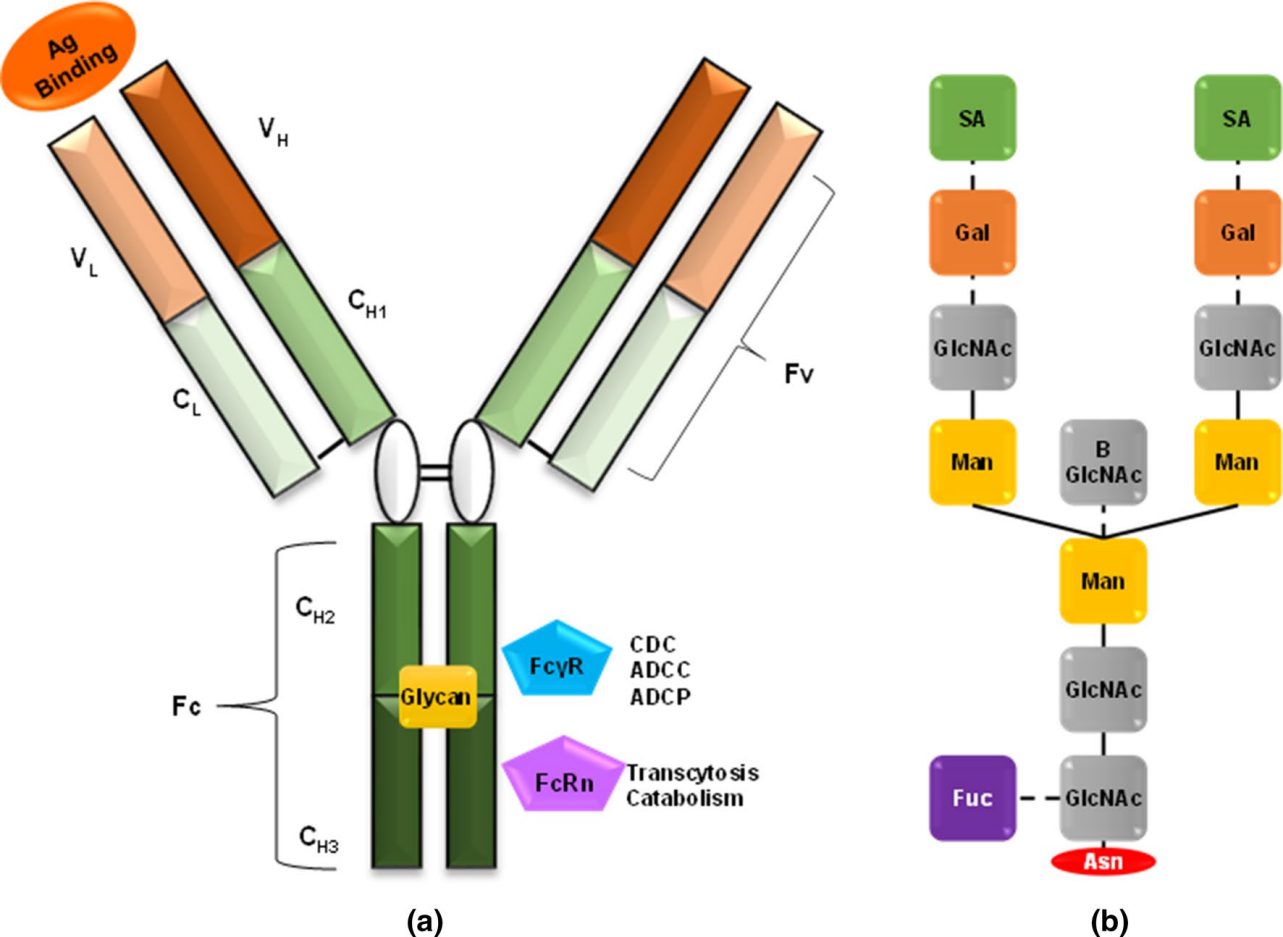
**a** Monoclonal antibody structure with binding site for antigen, FcγR and FcRn receptor as well as glycosylation sites (Glycan); *Ag* antigen, *CDC* complement-dependent cytotoxicity, *ADCC* antibody-dependent cell cytotoxicity, *ADCP* antibody-dependent cell phagocytosis, **b** glycosylation profile at N297 residue of the Fc region of antibodies. The *bold line* indicates core structures, and *dotted line* indicates variable structures. *Gal* galactose, *SA* sialic acid, *man* mannose, *GlcNAc N*-acetylglucosamine, *Fuc* fucose, *Asn* asparagine (N297)

Effector functions of mAbs and related products, such as antibody-dependent cell phagocytosis (ADCP), antibody-dependent cytotoxicity (ADCC), complement-dependent cytotoxicity (CDC) as well as evoking other cell-mediated immune responses, are modulated via the Fc region by interaction with FcγR receptors on different immune responsive cells (Fig. 2a) (Carter 2006). This also regulates the pharmacokinetics, transcytosis, catabolism and placental transfer of antibodies via the FcRn (neonatal Fc Receptor) as summarized in Table 2 (Roopenian and Akilesh 2007). Glycosylation at the Fc region occurs at N297 and consists of a core heptasaccharide region comprising mostly *N*-acetylglucosamine and mannose residues as well as the variable region as seen in Fig. 2b (Carter 2006). Modifying the Fc region either via amino acid substitution or by a change in glycosylation pattern has shown to change effector functionality. IgG1-based therapeutic antibodies have shown increased ADCC and ADCP activity with substitution at amino acid positions 298,333 and 334, whereas Otelixizumab has shown reduced ADCP and ADCC activity with an N297A substitutions(Shields et al. 2001; Bolt et al. 1993).The mammalian cell production systems could alter the glycoform, and this could either change the effector function-mediated therapeutic activity or induce immunogenic effects of mAbs (Jefferis 2009). Afucosylation and bisecting *N*-acetylglucosamine were reported for antibodies produced in CHO cells, and they were associated with reduced ADCC activity (Shields et al. 2002; Umaña et al. 1999). Galactosylation levels are important for different functions, such as transport of IgG molecules across placenta and complement activation. Mammalian cell lines generally produce hypogalactosylated products; however, if this hypogalactosylation is unintended, it could impact effector function. This has been demonstrated with Alemtuzumab and rituximab where the removal of galactose residues reduced complement activation (Raju and Jordan 2012; Boyd et al. 1995). Mammalian production systems can also add oligosaccharides not present in human system, such as addition of *N*-glycolylneuraminic acid by CHO, NS0 and Sp2/0 systems, which can be immunogenic (Jefferis 2014).

**Table 2:** IgG receptors and effector functions

	Function	Binding affinity		Expression	Important AA residues	Impact of glycosylation^b^
		IgG subclass	Ka (10^6^ M^−1^)			
C1q	CDC	** * *** **–**	**NA** *NA* ***NA*** NA	Present in serum	L235, D265, D270, K322, P329, P331, H433	Galactose: ↑ CDC; Mannose: ↓ CDC
FcγRI	Activation	*** **–** **** **	**65** **–** ***61*** 34	Monocytes, macrophages Dendritic Cells Neutrophils^**I**^ Mast Cells^**I**^	E233, L235, G236	Unclear
FcγRIIA (H131)	Activation	*** ** **** **	**5.2** *0.45* ***0.89*** 0.17	Monocytes, macrophages Dendritic Cells Neutrophils Mast Cells Basophils Eosinophils	L234, L235, G236, A327	Unclear
FcγRIIA (R131)		*** * **** **	**3.5** *0.10* ***0.91*** 0.21			
FcγRIIB/C	Inhibition	* **–** ** *	**0.12** *0.02* ***0.17*** 0.20	B cells Dendritic cells Basophils Monocytes^a^ Macrophages^a^ Neutrophils^a^	Unclear	Unclear
FcγRIIIA (F158)	Activation	** **–** **** **–**	**1.2** *0.03* ***7.7*** 0.20	Natural killer cells Monocytes Macrophages	E233, L234, L235G236	Mannose, Bisecting GlcNac:↑ ADCC; Sialic acid, fucose: ↓ ADCC
FcγRIIIA (V158)		*** * **** **	**2.0** *0.07* ***9.8*** 0.25			
FcγRIIIB	Unclear	*** **–** **** **–**	**0.2** **–** ***1.1*** **–**	Neutrophils Basophils	L234, L235G236, G237, P238	Unclear
FcRn	Transcytosis Catabolism Antigen uptake	*** *** **/**** ***	**80** *NA* ***NA*** NA	Monocytes, macrophages, Dendritic Cells Neutrophils Endothelium Syncytiotrophoblast	H433, N434, H435, Y436	Galactose, Mannose, GlcNAc: ↑ Clearance

Bold: IgG1, italic IgG2

Bolditalic: IgG3

Underline: IgG4

*NA* not applicable, *AA* amino acid

**** Very high affinity

*** High affinity

** Moderate affinity

* Low affinity;—no binding

^I^Inducible expression

^a^Low percentages

^b^Liu (2015)

Although the trends seem to be in favour of biopharmaceutical development, the growth rates have not yet reached their full potential due to financial and technical complexities involved in early stages of research and development and preclinical testing as described in the following sections. The comprehensive costs of developing a new drug amount to $2.8 billion (Pharma 2014). Studies done over the past decade show that nearly 90 % of drugs failed in clinical development (66 % in Phase I and 30 % in Phase II) and this high attrition rate is the major contributing factor to the exorbitant cost of new drug development (Hay et al. 2014; Kola and Landis 2004; Paul et al. 2010). Thus, it is more beneficial to address attrition, as a 10–15 % decrease in attrition rate could reduce the cost of drug development by nearly 35 % (Paul et al. 2010). Recent studies reported that toxicity and lack of efficacy were the most important factors for high attrition rates in small molecule drug development (Waring et al. 2015). Unlike conventional drugs which mainly revolve around small molecule chemistry, biological drugs are far more complex to produce and characterize as they are 200–1000× larger, structurally more complex and highly sensitive to their manufacturing conditions. The costs involved in development and production of biopharmaceutical entities are 1.5–2.5× higher than that of small molecule drugs (Blackstone and Fuhr 2007). With nearly 80 % of biological drugs failing in clinical development mainly due to lack of efficacy and safety, there arises an urgent need for smarter preclinical development. This requires better product understanding, i.e. examining characteristics which contribute to product quality such as biological activity, affinity, pharmacology, toxicity, immunogenicity, thus leading to early prediction of success/failure. Improved product understanding and rapid screening of potential drug candidates by utilizing different in vitro and in silico methods to predict efficacy and safety techniques would lead to better preclinical design.

### In vitro systems for toxicity testing

The general in vitro toxicity testing panel includes cellular, biochemical and molecular assays to study cytotoxicity, reactive oxygen species production as well as specialized toxicity effects including genotoxicity, hepatotoxicity, immunotoxicity to name a few. They are assessed via standard, specialized or target organ cell-based assays. Techniques such as WST, MTT, MTS, BrDu and Alamar blue are commonly used to asses basal cytotoxic or direct effect on cell proliferation, whereas Annexin V/Propidium iodide staining can help distinguish between necrotic and apoptotic events. Mitochondrial damage can be assessed by mitochondrial membrane potential assays and luminescent cell viability assays that quantify ATP. Protein marker-based techniques, such as assessing caspase cleavage via flow cytometry or western blotting techniques, can also be used to understand the mode of action of particular compounds. Reactive oxygen species production leads to oxidative stress, and this can also lead to cellular damage. There are different dyes, such as fluorescent and bioluminescent dyes, that can be utilized to study this effect. For gauging specialized toxicity effects, different types of biochemical, molecular and mode of action-based endpoints can be utilized. In vitro experimental data when combined with physicochemical properties and absorption, distribution, metabolism and elimination (ADME) characteristics help establish physiologically based pharmacokinetic (PBPK) and partitioning models (based on fundamental thermodynamic principles). Metabolism of parent compound, toxicity and likelihood of metabolites also allow for a more robust model to be developed as they help to take into account biotransformation and bioavailability. The above information helps to identify the doses and the class of compounds that have to be further tested in in vivo tests as specified by OECD guidelines for toxicity testing.

Monoclonal antibodies evoke an effector response mainly via antibody-dependent cytotoxicity, phagocytosis and complement-dependent cytotoxicity for eliminating tumour target cells (Kindt et al. 2007). For testing the biological activity of mAb-based therapeutics in vitro, the target cell line is cocultured with the molecule as well as effector cells derived either from PBMCs in human blood or cultured effector cells in a defined target to effector ratio (Golay et al. 2013). These effects can be studied by techniques which involve loading target cells with fluorescent membrane permeable dyes that are released upon target cell lysis. To assess mast cell degranulation, in vitro systems are incubated with drug of interest, and endpoints like histamine are then measured via spectroscopy or flow cytometry (Demo et al. 1999). Alternatively specific biomarkers like complement fragments can be used to detect specific events such as complement activation (Golay and Introna 2012). Cytokine release assays provide information about the extent and the kind of pro-inflammatory cytokine release. This is often assessed by introducing the monoclonal antibody to human lymphocytes and then assessing the supernatant for different types of cytokines, and this assay can often be performed in a multiplex format with flow cytometer analysis (Lash et al. 2006). A cytokine storm is a life-threatening adverse effect induced by monoclonal antibodies such as in the case of TGN1412 (Suntharalingam et al. 2006). Animal models utilized for assessing immunotoxicity involve lymph node proliferation assay, local lymph node assay and more recently the mouse drug allergy model though the predictive ability of these in vivo models have not been well characterized or validated (Whritenour et al. 2016). For assessing specialized toxicity assays, specific endpoints or biomarkers can be studied. Drug induced liver injury, liver enzyme inhibition or induction (particularly cytochromes 450, flavin monooxygenases and numerous others), change in human pregnane X Receptor activity as well as drug transporter activities for hepatotoxicity; Ames test for mutagenicity, in vitro single cell electrophoresis (comet) assay and DNA-based dyes for genotoxicity; human ether-related à-gogo gene related (hERG) assays, prolongation of QT interval, patch clamp assay, embryonic stem cell differentiation assay for cardiotoxicity and so on are examples used in small molecule drug development (Ekins 2014).

These issues regarding pharmacodynamics, selection of model organism, route of administration, dose, metabolism, toxicity studies have been addressed by the ICH Safety Pharmacology guideline *S6 (R1) Preclinical Safety Evaluation of Biotechnology*-*Derived Pharmaceuticals*. The safety pharmacology of mAbs, however, cannot be optimally assessed by standard toxicological assays alone (Cavagnaro 2002; Guideline 1997).

### In silico tools for predictive toxicology

Computational toxicology tools could substantially aid in safety pharmacology testing of monoclonal antibody-derived therapeutics as they impart elements of automation, consistency and reliability to standard toxicological assays. There are a multitude of advantages offered by computational toxicology methods. They help to realize the 3R principle, i.e. replacement, reduction and refinement, by reducing the number of experimental animals used in drug safety testing. They also address the practical and economical concern of industries by providing a rapid and cost-effective way for safety testing of novel drug molecules. This in turn helps to cut down attrition rates and thus reduce the financial burden on the discovery and the development of new drugs. Furthermore, computational toxicology methods help to prioritize testing of those compounds which could be associated with toxic hazards by virtue of a problematic chemical space. This could be by means of structural similarity, indiscriminate interaction with closely related pharmacological targets and/or off target effect or other molecular events which are adaptable to in silico methods. Computational toxicology methods also prove useful when animal studies do not adequately represent the fate of drugs in humans (Ekins 2014; Cronin and Madden 2010; Greene and Pennie 2015; Wilson 2011).

Though these in vitro and in silico methods, such as physiologically based pharmacokinetic (PBPK) modelling and qualitative/quantitative structure–activity relationships (QSAR), are extensively used for predicting biological activity as well as toxicity during small molecule drug development (Table 3), their full potential has not been utilized for biological drug development.

**Table 3:** Comprehensive overview of in silico prediction tools for assessing toxicology

Name	Particulars	Accessibility	Owned by
ACD ToxSuite	Molecular fragment QSAR and knowledge expert system, (Perceptra platform) employing machine learning^a,h,i,j,k,l,m,r,s^	Commercial	ACD Labs, Pharma algorithms
Admensa interactive™	QSAR-based system^h,k,l^	Commercial	Inpharmatica Ltd.
ADMET™ predictor	QSAR-based expert system and machine learning^b,c,d,e,f,j,k^	Commercial	Stimulation Plus Inc.
ADMEWORKS Predictor	QSAR,QSPR-based expert system^a,b,l^	Commercial	Fujitsu, Poland
AIM	Category formation and read across	Free	US EPA
BfR decision support system	SAR and physicochemical exclusion rule-based system. Employs concordance decision tree approach^d,i,o^	Free	German Federal Institute for Risk Assessment
BioEpisteme	Molecular descriptor QSAR^b,h,k,n^	Commercial	Prous Institute for Biomedical Research, Spain
Bio-loom	QSAR database CLOGP, CMR^h,j^	Commercial	Biobyte
CAESAR	QSAR-based expert systems based on Dragon descriptors and Multivariate approaches^a,b,d,e^	Free	EU
CaseUltra (MC4PC)	Molecular fragment QSAR-based expert system using machine learning^a,b,c,d,I,j,k^	Commercial	MultiCASE Inc.
Cerius^2^/Material Studio	Molecular modelling software^k,l^	Commercial	Accelrys Inc.
COMPACT	SAR and knowledge-based system employs molecular orbital descriptors^a,b,c,k^	Free	US NTP
CSgenoTOX	QSAR-based system and machine learning(ANN)^a^	Commercial	ChemSilico
DEREK NEXUS	SAR knowledge-based expert system^a,b,c,d,e^	Commercial	Lhasa Ltd.
HazardExpert (ToxAlert)	QSAR knowledge-based expert system^a,b,d,e,n,o,p^	Commercial	Compudrug Inc.
Insilicofirst	Common user interface expert system	Commercial	Lhasa Ltd., Leadscope, Multicase, MN GmbH
KNIME^®^	QSAR workflow tool	Open	KNIME.com
LAZAR	KNN approach (machine learning)^a,b,k^	Open source	In silico toxicology GmbH
Leadscope model applier	QSAR and expert rule-based knowledge system^b,c,e,g,h,k,n^	Commercial	Leadscope Inc.
MDL QSAR	Molecular descriptor QSAR, QSPR, multivariate approaches^a,b,h,j^	Commercial	Symyx - MDL, Inc.
Molcode toolbox	QSAR-based prediction tool^a,b,d,i,j^	Commercial	Molcode Ltd.
OECD QSAR toolbox	Category formation and read across, QSAR for multiple endpoints	Free	OECD
Oncologic™	SAR rule-based expert system. Employs hierarchical decision tree approach^b^	Free	US EPA
PASS	SAR-based expert system using biological activity spectra and MNA^b,j,o,r^	Free	geneXplain GmbH
Pre ADMET	QSAR-based system and machine learning^a,b,l^	Commercial	BMDRC Korea
QikProp	QSAR-based expert system^h,l^	Commercial	Schrödinger Inc.
q-TOX	Knowledge-based expert system^f,h,j,k,m,n^	Commercial	Quantum pharmaceuticals
Sarah nexus	Statistical software tool^a^	Commercial	Lhasa Ltd.
StarDrop	QSAR-based expert system^h^	Commercial	Optibrium Ltd.
T.E.S.T	QSAR-based expert system and machine learning^g,j^	Free	US EPA
TerraQSAR	Molecular fragment QSAR-based expert system. Employs probabilistic neural networks^d,g,j,o^	Commercial	TerraBase Inc.
TIMES	Structural alerts and COREPA software-based hybrid expert system^a,d,g^	Commercial	Bourgas University, Bulgaria
TOPKAT	QSAR, SAR, QSTR-based expert system using Bayesian classification and partial least square regression models^b,c,d,e,i,j,k,q,^	Commercial	BIOVIA Discovery Studio^®^
ToxMatch	Category formation and read across^d^	Free	Ideaconsult Ltd.
ToxTree	Category formation and read across^a,b,c,d,i,l^	Free	Ideaconsult Ltd.
ToxWiz	Knowledge base expert system	Commercial	Cambridge cell networks

*AIM* analog identification methodology *US EPA* united states environmental protection agency, *FDA* food and drugs administration, *NTP* national toxicology program *EU* European Union, *QSAR* quantitative structure—activity relationships, *QSPR* quantitative structure—property relationship, *QSTR* quantitative—structure toxicity relationship, *TOPKAT* toxicity prediction by computer assisted technology, *PASS* prediction of biological activity spectra for substances, *CAESAR* computer assisted evaluation of industrial chemical substances according to regulations, *T.E.S.T* toxicity estimation software tool, *COMPACT* computer-optimized parametric analysis of chemical toxicity, *LAZAR* lazy structure–activity relationships, *TIMES* tissue metabolism simulator, *ADMET* absorption, distribution, metabolism, excretion, toxicity, *MNA* multilevel neighbourhood of atoms, *COREPA* common reactivity pattern approach, *ANN* artificial neural networks

^a^Mutagenicity, ^b^ carcinogenicity, ^c^ genotoxicity, ^d^ dermal toxicity, ^e^ developmental toxicity, ^f^ pulmonary toxicity, ^g^ reproductive toxicity, ^h^ cardiotoxicity, ^I^ Occular toxicity, ^j^ acute toxicity, ^k^ hepatotoxicity, ^l^ absorption, distribution, metabolism, excretion, ^m^ renal toxicity, ^n^ neurotoxicity, ^o^ immunotoxicity, ^p^ cytotoxicity, ^q^ chronic toxicity, ^r^ haemotoxicity, ^s^ gastrointestinal toxicity

### Predictive model development

**Fig. 3:**
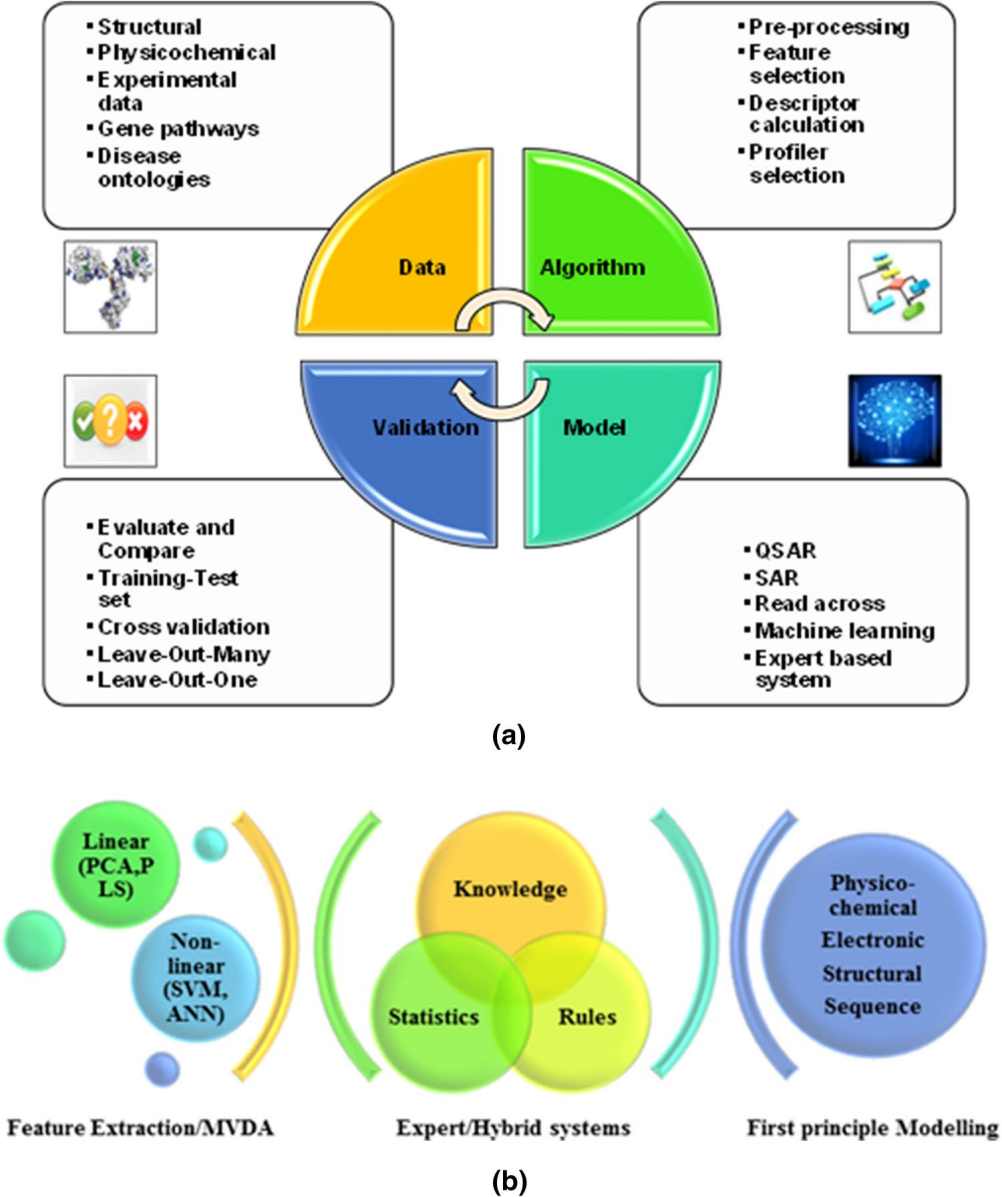
**a** Computational toxicology model development workflow, **b** techniques involved in different types of predictive models

From the different in silico tools listed in Table 3, a summarized workflow for predictive toxicology model development is depicted in Fig. 3a. The main question to consider while developing a computational model is what can be modelled? The starting point of model development is data which can be of different types such as numeric, categorical, discrete or continuous and can be acquired from different sources like experiments, structures, physicochemical properties and so on. Algorithms are then required to preprocess these data as well as for feature extraction. This is mainly for selecting the inputs and outputs of models as well as to convert raw data into parameters that can be modelled mathematically, i.e. profilers or descriptors. Different linear and nonlinear mathematical techniques can be used for associating these descriptors to an adverse effect or toxicity by means of statistics, rules, multivariate data analysis and/or expert knowledge thus leading to development of a predictive model as shown in Fig. 3b. The resulting model must be validated to ensure non-discriminatory comparison with other existing models. Several factors would have to be taken into consideration while selecting a software platform/tool such as availability, accessibility, user expertise levels, transparency of algorithm and knowledge base, choice and complexity of methodology and inclusion of mechanistic elucidation. Performance would depend on choice of measures for robustness and goodness of fit as well as validation parameters and methods chosen. Some of these aspects are described in detail in the following sections keeping in mind the proteinaceous nature of mAbs.

### Databases

A number of databases have been utilized for developing predictive toxicology models during small molecule development such as Open TG GATEs, Pharmapendium, Drugmatrix^®^ and ToxFX^®^ (Greene and Pennie 2015). Databases containing information about mAbs and derived therapeutics are being developed extensively, and the IMGT mAb database is particularly noteworthy in this regard as it provides comprehensive information on structure, primary sequences, developmental status, targets as well as documents relating to approval for more than 589 entities (Lefranc et al. 2015). Sources like Drug Bank, patents, FDA documents and UniProt could yield useful information regarding sequences of mAbs, whereas Protein Data Bank (PDB) could provide structural information. The choice of a dataset for training model impacts its performance as studies have frequently indicated the discrepancies between public and proprietary datasets, i.e. performance of a model developed on public datasets is lower when applied on a proprietary dataset (Greene and Pennie 2015).

### Descriptor generation and model development

Multivariate and statistical data analysis techniques have further allowed for rapid and easier descriptor calculation and model development. For proteins, the primary amino acid sequence and in some cases the 3D structure form the basis of generating different physicochemical, thermodynamic and topographic indices where the physicochemical and structural characteristics of amino acids are utilized to derive descriptors. These include principal component analysis-derived descriptors such as z scales and *T*-scales; 3D structure-based ones such as isotropic surface area and electronic charge index; atomic charge density-derived ones such as transferable atomic equivalent, to name a few (van Westen et al. 2013a, b). Several machine learning and statistical methodologies, such as support vector machines (SVM), artificial neural networks (ANNs), *k*-nearest neighbor approach (kNN), decision forest approach, Naïve Bayes, C4.5 decision tree, Bayesian models, random forest approaches, recursive partitioning, multiple linear regression (MLR), discriminant analysis (DA) and self-organizing maps (SOM), have been used to predict hepatotoxicity, genotoxicity, cardiotoxicity and renal toxicity of small molecules (Ekins 2014; Greene and Pennie 2015; Wilson 2011; Hardy et al. 2010). They can be used to build standalone inference-based models or combined with quantitative structure–activity relationship modelling.

### Models

Quantitative structure–activity relationships (QSAR) approach is based on connecting an activity, in particular toxicity (QSTR) or any other property (QSPR), to descriptors which can be derived from physicochemical, structural, electronic or steric parameters (Hansch et al. 1995). QSAR methodology works best when the biological activity in question is based on a single endpoint or a simplistic mechanism of action. The development of QSAR models has been supported extensively by workflow tools, QSAR databases as well as uniform reporting and summarizing formats. Expert/Hybrid systems are extension of QSAR models, and they can be based on rules, knowledge or statistics as well as a combination of two or more approaches. The multivariate techniques used can either be linear, such as principal component analysis (PCA) or partial least square regression (PLS) used in TOPKAT, or nonlinear techniques, such as ANNs, used in CSgenoTox (Cronin and Madden 2010). Knowledge-based expert systems have incorporated a more mechanistic basis to their predictive tools (Cronin and Madden 2010). QSAM (quantitative sequence activity modelling) is another paradigm of QSAR modelling which is being used extensively for protein-based predictive models. Angiotensin-converting enzyme (ACE)-inhibitory peptides were screened based on models generated using PLS, MLR and most recently ANN (Zhou et al. 2008). PLS, SVM and HM-based models have been used with smaller peptides (9 amino acids residues) for predicting binding affinity with Class I Major Histocompatibility Complex (Zhao et al. 2007). Proteochemometric modelling is an extension of QSAR that uses multiplication of ligand and protein descriptors (MLPD) to include interaction space information in addition to protein and ligand descriptors (Qiu et al. 2016).

The advantages of QSAR-based expert systems are that they are rapid, well developed and regularly updated. The disadvantages are that the datasets, algorithms and knowledge base are usually not transparent. Most of the tools are commercial and use proprietary datasets. Due to the high level of automation, there is a possibility of losing the mechanistic understanding of action.

In addition to the models mentioned above, significant advances have been made with regard to ADME models as understanding the ADME characteristics of molecules is very important in assessing their bioavailability. A target mediate drug disposition-based pharmacokinetic model has been developed from preclinical data for predicting pharmacokinetics of mAbs within the human system which could aid in clinical designs (Luu et al. 2012). There have been several machine learning techniques that have been employed in skin absorption and metabolizing studies which enable to predict the extent of toxicity caused by compounds (Ashrafi et al. 2015; Moore et al. 2014). It is also worthwhile to mention that the latest techniques seem to revolve around consensus modelling where the outputs from different predictive models are averaged or inferred by several approaches, for example, leverage-weighted means (Cronin and Madden 2010). The success of these models, however, has been debatable as some report better predictivity, while others report no significant benefits when compared to single models (Hewitt et al. 2007).

### Validation

Models are assessed for specificity, sensitivity and concordance based on either a different dataset typically referred to as the test set or by other appropriate means of validation. Internal validation procedures implemented include cross-validation (leave out one and/or leave out many) and bootstrapping. External and independent validation strategies can also be used such as testing the model with new experimental data. The predictive ability can be quantified using different parameters like root-mean-square error (RMSE), determination coefficient (*R* ^2^) and predictive squared correlation coefficient (*Q* ^2^) for QSAR model, and these have been evaluated in previous studies (Abshear et al. 2006; Consonni et al. 2009).

## Discussion: status Quo and scope for mAb-based application

Different approaches have to be adopted for safety evaluation of monoclonal antibody-derived therapeutics when compared to small molecule drugs owing to innate differences like species specificity, degradation, increased half-life, complex dose–response relationship, interaction, lack of generic testing material, pleiotropic and synergistic mechanisms to name a few (Cavagnaro 2002).

Whether it is for assessing preclinical safety or for rapid screening, in vivo systems are not the most suitable models for studying the effects of monoclonal antibody-based therapeutics. The rationale behind using in vivo studies in preclinical safety testing is that the indirect immune-mediated response induced by the antibody as well as the magnitude of the effect cannot be gauged via standard in vitro tests. However, species specificity still remains the main obstacle. Studying the effector function becomes difficult due to differences in the FcγR receptors structure and affinity, complement system response and absence of target antigen (Golay and Introna 2012). Presence, number, interactions as well as distribution of target antigen also play an important role in assessing the biological activity of monoclonal antibodies (Golay et al. 2001). Attempts have been made to solve this problem by different strategies, such as knockout mice that lack mouse FcγR, transgenic mice expressing human FcγR, generating xenografts with human antigen in mouse cell lines, using completely mouse systems and using primate models such as rhesus monkey (Golay and Introna 2012; Barouch et al. 2013; Bournazos et al. 2014; Strasser et al. 2013). Animal testing is also expensive, sample size dependent and resource intensive. The main bottleneck in using in vitro systems for assessing the toxicity of mAbs is that the effector cells have to be coincubated or cocultured with the cell line of interest. The sensitivity and specificity of these assays depend on several factors which have to be optimized, such as cell density, incubation times as well as the choice of system and assay endpoint. The innate complexity, diversity and size of mAbs-based therapeutic as well as their diverse mechanisms of actions that involve many pathways exacerbate the need for carefully designed in vitro systems that take into account all of the above factors. In standard cytokine release assays, the mAbs bind to receptors all over the cell which is not an accurate representation of the human systems where cytokine release is sometimes dependent on localized receptor interaction (Stebbings et al. 2007). Sophisticated analytical techniques used in studying the endpoints of these assays have to be carefully assessed for resolution as well as sensitivity in detecting events as they can be prone to artefacts owing to nature of assay in question as well as the size of biological molecules. Artefacts can arise while using flow cytometry techniques due to homotypic adhesion as demonstrated with anti CD20 antibodies monoclonal antibodies (Golay et al. 2010). New generation preclinical safety testing tools would have to be high-throughput, rapid and cost-effective to meet the accelerated growth of the biopharmaceutical market. They also need to be highly reproducible and be fairly predictive to allow for rapid screening facilitating reliable selection of new compounds at initial stages thus saving time and money to allow more focus on drug development for rare diseases. They would also provide an alternative to animal testing considering the various drawbacks of in vivo systems as seen in the case of TGN1412. In vitro systems have now evolved from 2D cocultures to 3D spheroidal cocultures, organs on chips as well as whole blood systems to better mimic the responses that could be produced in a human system (Whritenour et al. 2016). Immunotoxicogenomics and expression profiling of both in vivo and in vitro systems are being used to identify pathways, mechanism of action as well as biomarkers for study of delayed hypersensitivity reactions (Shao et al. 2014). These advancements may contribute to better designed preclinical testing strategies for monoclonal antibody-derived therapeutics.

Appropriate and relevant experimental studies are of paramount importance in non-clinical safety testing as they also contribute to good datasets which can then be modelled. Most of the models are developed based on public datasets and fail to perform adequately when tested with proprietary datasets. The highly competitive nature of the biopharmaceutical industry makes information access very difficult. There are also difficulties in feature extraction for biological molecules owing to their complexity and size. The applicability of such modelling techniques in rapid screening depends on the experimental set-up as well as on identifying and forming sensible profilers and descriptors. Like all proteins, the primary sequence provides a wealth of information for mAbs. However, there is high degree of sequence similarity, especially in the Fc region, and this would mean that appropriate techniques such as benchmarking would have to be incorporated to select relevant descriptor sets (van Westen et al. 2013a, b). Descriptor for proteins molecules can be generated by different software such as PseAAC, Protein Recon, PROFEAT and ProtDCal, of which ProtDCal, a freely available tool with a friendly graphical user interface, has the capacity to generate a higher number of non-redundant of molecular descriptors for proteins from FASTA or PDB files (Ruiz-Blanco et al. 2015). Another possible concern is that primary sequence-based descriptors do not take into account neither interactions between amino acid residues nor the antibody-antigen and antibody-receptor interaction space. There are different modelling platforms for predicting antibody structures from primary sequences such as PIGS(Prediction of Immunoglobulin Structures), Rosetta antibody, Web Antibody Modelling (WAM) and Abysis databases among which PIGS performs better (Marcatili et al. 2014). RCSB integrates different bioinformatics and structural tools for comparison of primary and secondary structures. Advances made in PCM techniques include a new descriptor for antigen–antibody interaction called epitope–paratope interaction fingerprint (EPIF) which tries to address the higher time-complexity of MLPD, thus allowing for simplification the antigen–antibody interaction term (Qiu et al. 2015, 2016). Platforms like proABC, ABangle and LYRA allow for modelling antigen–antibody interactions, orientation of variable chain and lymphocyte receptor, respectively (Klausen et al. 2015; Olimpieri et al. 2013; Dunbar et al. 2013). Physicochemical characteristics of mAbs will influence PK/PD properties (increased binding to serum proteins and increased half-life) which affects ADME characteristics thus impacting bioavailability and biological activity. Glycosylation is another aspect that has to be taken into consideration as change in glycosylation pattern could affect functionality as well as impact PK/PD characteristics of mAbs (Liu 2015). Successful attempts have been made from a bioengineering point of view to investigate the effects of the production process on glycosylation profiles of monoclonal antibodies by using multivariate techniques, such as principal component analysis, partial least squares and parallel factor analysis (Green and Glassey 2015; Glassey 2012). Glycoengineered antibodies were produced by CHO cells with higher glycosyltransferase which enabled the production of engineered antibodies with the N-acetylglucosamine profiles required to achieve higher neutrophil-mediated phagocytosis activity and thus greater efficacy in killing tumour cells (Umaña et al. 1999; Golay et al. 2013). Indeed, engineered glycoforms of anti-CD20 antibodies, such as obinutuzumab and rituximab, have sevenfold higher binding affinity to neutrophils and thus an increased neutrophil-mediated phagocytosis-based killing of tumour cells (Golay et al. 2013). The challenge would then be to associate these attributes to potential adverse effects which will then allow for development of predictive toxicology models. Intricate algorithms would also be required for associating profilers and descriptors with synergistic endpoints of toxicity. Along with carefully designed experimental procedures, extensive expert knowledge would be required for such model development.

## Conclusion

Biopharmaceuticals have positively impacted the lives of millions. They have paved the way for personalized medicines, improve prognosis of cancer, genetic and immune disorders as well as breakthroughs in rare disease management. The advances made are, however, impeded by a lack of progress in bioprocess development strategies as well as increasing costs owing to attrition, wherein the lack of efficacy and safety accounts for nearly 60 % of all factors contributing to attrition (Kola and Landis 2004). This reiterates the need for carefully designed predictive models to assess the efficacy as well as toxicity of potential drug candidates at an early stage. A more effective, high-throughput rapid screening of candidates based on adverse effects is required at an early stage to filter out the number of candidates proceeding to clinical trials. A choice of appropriate in vivo systems should be in place along with better proof of concept studies as animal models are not representative of human systems for assessing the efficacy and safety of biopharmaceuticals in specialized therapy areas like oncology and immunology. Alternative approaches such as specialized in vitro toxicology tests, better biomarkers and *omics* approaches can be utilized for this purpose. In this regard, computational toxicology tools like expert/hybrid systems provide a powerful complement to in vitro systems as they will allow for development of automated and reliable models for predicting toxicity or adverse effect of monoclonal antibody therapeutics. In order to make these predictive platforms more robust, descriptor calculation, feature extraction, inclusion of pharmacokinetics and bioavailability characteristics, mechanistic understanding and multidisciplinary expert knowledge will be of paramount importance. This will pave way for the development of rapid bioprocess development strategies for faster development of effective and safe biopharmaceuticals and may in fact change the face of biopharmaceutical manufacturing as we see today.

## Electronic supplementary material

Below is the link to the electronic supplementary material.

Supplementary material 1 (DOCX 13 kb)
