# Supplementary material for: Applicability of predictive toxicology methods for monoclonal antibody therapeutics: status Quo and scope
Source: Arch Toxicol. 2016 Oct 20;91(4):1595–612. doi: 10.1007/s00204-016-1876-7 (PMC5364268; doi:10.1007/s00204-016-1876-7)
Supplement: Supplementary file 1 — Supplementary material 1 (DOCX 13 kb) [file 204_2016_1876_MOESM1_ESM.docx]

## **Supplementary file 1: Adverse effects of mAbs categorised into specialised toxicities.**

**Immunotoxicity :**

Infusion reactions (acute, severe) hypersensitivity, Immunogenicity, anaphylaxis (0.1%), Churg-Strauss syndrome, acute infusion reactions, cytokine release syndrome, immunosuppression, IgE against oligosaccharide and HAMA, immune haemolytic anaemia, Immune thrombocytopenia, Serum Sickness.

**Infection:**

Upper respiratory tract infections Progressive multifocal leukoencephalopathy Hepatitis B reactivation, serious opportunistic viral and/or bacterial infection, Meningococcal and Neisseria infection, tuberculosis reactivation, osteomyelitis.

**Haemotoxicity:**

Intravascular haemolysis, haemolytic anaemia, thrombocytopenia, haemorrhage, arterial and venous thromboembolic events, pancytopenia, lymphopenia, leukopenia, neutropenia, increased risk of bleeding, osteonecrosis, arterial thromboembolism, Epistaxis.

**Cardiotoxicity:**

Transient hypotension, cardiac arrhythmias, severe hypertension, congestive heart failure, cardiomyopathy, pericarditis, myocardial infarction, ischaemic attack.

**Hepatotoxicity:**

Elevated liver transamines, abnormal liver function, lipid deregulation, neutropenia.

**Gastrointestinal Toxicity:**

Diarrhoea nausea vomiting, gastrointestinal perforation, bowel obstruction, enterocolitis.

**Pulmonary toxicity:**

Bronchospasm, interstitial lung disease, pulmonary fibrosis, pneumonitis, pulmonary embolism, pneumothorax.

**Ocular Toxicity:**

Conjunctival haemorrhage, intraocular inflammation, increased intraocular pressure, retinal detachment, endophthalmitis. Uveitis.

**Dermal toxicity:**

Injection site reaction, severe mucocutaneous reactions, skin rashes and reactions, urticarial, angioedema.

**Renal Toxicity:**

Nephritis, proteinuria, tubular damage, hypophosphatemia, hypomagnesemia, renal failure, pyelonephritis.

**Neurotoxicity:**

Guillain–Barré syndrome, encephalitis, meningitis, neuropathy, neurocognitive disorders.

**Reproductive/Developmental Toxicity:**

Birth defects, Embryo/foetal mortality.

**Others:**

Fever, headache, cough, secondary malignancies, wound dehiscence, thyroid disorders,
